# Proton pump inhibitors act synergistically with fluconazole against resistant *Candida albicans*

**DOI:** 10.1038/s41598-019-57174-4

**Published:** 2020-01-16

**Authors:** Mengjiao Lu, Haiying Yan, Cuixiang Yu, Lei Yuan, Shujuan Sun

**Affiliations:** 1 Department of Pharmacy, Shandong Provincial Qianfoshan Hospital, Shandong University, Ji’nan, 250014 Shandong Province P.R. China; 2 Department of Pharmacy, Tianjin Baodi Hospital, Baodi Clinical College of Tianjin Medical University, Tianjin, 301800 P.R. China; 3 Department of Pharmacy, Shandong Provincial Qianfoshan Hospital, the First Hospital Affiliated with Shandong First Medical University, Ji’nan, 250014 Shandong Province P.R. China; 4 Respiration Medicine, Shandong Provincial Qianfoshan Hospital, the First Hospital Affiliated with Shandong First Medical University, Ji’nan, 250014 Shandong Province P.R. China

**Keywords:** Antifungal agents, Fungi

## Abstract

The incidence of resistant *Candida* isolates, especially *Candida albicans*, has increased continuously. To overcome the resistance, research on antifungal agent sensitizers has attracted considerable attention. Omeprazole and lansoprazole were found to inhibit the growth of sensitive *C*. *albicans* and hyphae formation in a high dose, respectively. This study aimed to determine the interactions of common clinically proton pump inhibitors (PPIs) and fluconazole both *in vitro* and *in vivo* and to further explore the possible mechanisms. *In vitro*, the tested PPIs all acted synergistically with fluconazole against both resistant *C*. *albicans* planktonic cells and biofilms preformed for ≤12 h with the minimum inhibitory concentration of fluconazole decreased from >512 μg/mL to 1–4 μg/mL. *In vivo*, PPIs plus fluconazole prolonged the survival rate of infected *Galleria mellonella* larvae by two-fold compared with that for the fluconazole monotherapy group and significantly reduced the tissue damage of infected larvae. Mechanism studies showed that PPIs significantly suppressed efflux pump activity, which is the common resistance mechanism of *C*. *albicans*, and significantly inhibited the virulence factors: phospholipase activity and morphology switching. These findings will provide new insights into antifungal agent discovery and potential approaches for the treatment of candidiasis caused by resistant *C*. *albicans*.

## Introduction

The incidence of invasive fungal infections has increased continuously, especially those caused by *Candida* species^1,2^. *Candida* species can cause superficial infection of the skin, mouth, or mucous membranes and can also cause invasive infection, such as candidemia and biofilm-related infection^3^. In *Candida* infections, *C*. *albicans* is still the most commonly isolated strain. Data from the Prospective Antifungal Therapy Alliance registry showed that among the 7526 fungi isolated from 6807 invasive fungal infections, the isolation rate of *Candida* species was highest (n = 5526, 73.4%), and *C*. *albicans* accounted for 47.8% of its isolation rate^4^. Owing to its great efficacy and low toxicity, fluconazole (FLC) has been extensively used in clinical practice to prevent and treat candidiasis. However, along with the increased in frequency of infections and extensive use of FLC, drug-resistant strains have frequently emerged^5,6^. To overcome fungal resistance, research on antifungal sensitizers has attracted considerable attention.

Proton pump inhibitors (PPIs) inhibit the H^+^/K^+^-ATPase in the cell membrane and have become the first choice in the treatment of acid-related diseases^7,8^. PPIs with a wide range of clinical applications include omeprazole (OME), lansoprazole (LAN), pantoprazole (PTP), rabeprazole (RAB), esomeprazole (ESO) and ilaprazole (ILA). OME was found to cure acute oesophageal necrosis and candidal oesophageal when it was combined with FLC in the clinic^9–11^. Studies on the antifungal activity of PPIs found that LAN and OME at a dose of >600 µg/mL could inhibit the growth of sensitive *C*. *albicans* and hyphae formation, respectively^12,13^. In addition, although some studies showed that PPIs combined with fluconazole *in vitro* have no synergistic effects against sensitive *C*. *albicans*^13–15^, other studies found that BM2, a D-octapeptide inhibitor of the plasma membrane proton pump, enhanced the efficacy of FLC against resistant *C*. *albicans* and *Candida dubliniensis*^16,17^. However, no study has reported the interaction of commonly used PPIs and FLC against resistant *C*. *albicans*. In this study, we evaluated *in vitro* and *in vivo* interactions of PPIs combined with FLC against resistant *C*. *albicans*, and the underlying mechanism of the interactions of PPIs and FLC was further explored.

In the present study, the *in vitro* antifungal activity of PPIs alone or combined with FLC was determined by the microdilution method, and an XTT assay was conducted to evaluate the antibiofilm effects of the drug combination. In addition, the *in vivo* interaction of the drug combination was evaluated by the establishment of a *G*. *mellonella* larvae infection model. Of note, with OME and RAB as representative PPI drugs, synergistic mechanisms were evaluated by assessing extracellular phospholipase activity, morphology switching and the efflux pump activity.

## Results

### PPIs acted synergistically with FLC against resistant *C*. *albicans in vitro*

**Figure 1:**
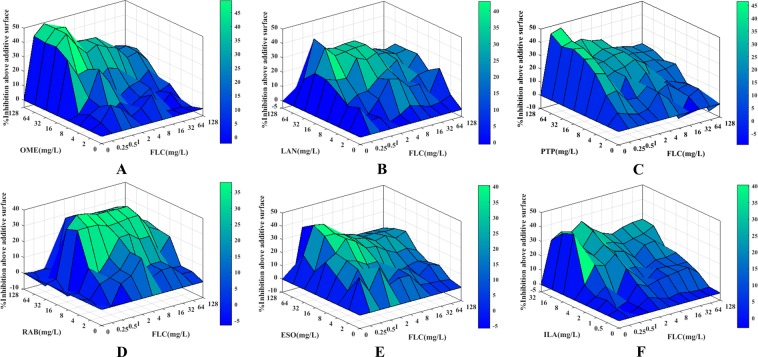
Three-dimensional model of PPIs combined with FLC against CA10 *in vitro*. (**A**–**F**) show the three-dimensional model of OME, LAN, PTP, RAB, ESO and ILA combined with FLC, respectively. The Δ*E* values are depicted on the *z*-axis, and the peaks above the 0 plane indicate synergistic combinations, whereas the peaks below the 0 plane indicate antagonistic combinations.

The minimal inhibitory concentrations (MICs) of PPIs and FLC against resistant *C*. *albicans* are listed in Table 1. The MIC of FLC was all >512 μg/mL for all tested *C*. *albicans* strains, indicating strong resistance of these *C*. *albicans* strains. The MICs of RAB, ILA and the others were 128–512 μg/mL, >256 μg/mL and >512 μg/mL, respectively, showing that RAB possessed a weak intrinsic antifungal activity and a very limited intrinsic efficacy for the other PPIs. However, when used in combination with FLC, PPIs could significantly decrease the MICs of FLC from >512 μg/mL to 0.5–4 μg/mL, indicating a significantly increased sensitivity of resistant *C*. *albicans* to FLC caused by PPIs. Of these six PPIs, when the MIC of FLC was decreased to ≤2 μg/mL, the concentrations of PPIs required were 8 µg/mL for ILA, 16–32 µg/mL for RAB, 16–32 µg/mL for LAN, 32 µg/mL for OME, 32 µg/mL for ESO and 32–64 µg/mL for PTP. Moreover, the FICI values obtained from the FICI model were 0.06 for OME and ESO, 0.03–0.06 for LAN and ILA, 0.06–0.13 for PTP and 0.04–0.25 for RAB. The FICI values were all <0.5, showing a strong synergism induced by PPIs plus FLC. Additionally, this synergistic effect was demonstrated by another evaluation model (Table 1, Fig. 1), with the ΣSYN values all >800%, far more than 200%, indicating that PPIs in combination with FLC exerted synergistic inhibitory effects on the growth of resistant *C*. *albicans*.

**Table 1:** *In vitro* interaction of PPIs with FLC against resistant *C*. *albicans*.

PPIs^a^	Strains^b^	MIC_80_ (µg/mL)^c^				FICI model		Δ*E* model		
		Alone		Combined		FICI^c^	IN^a^	ΣSYN (%)c	ΣANT (%)c	IN^a^
		PPIs	FLC	PPIs	FLC					
OME	CA10	>512	>512	32	0.5	0.06	SYN	1469.95	−5.31	SYN
	CA16	>512	>512	32	0.5	0.06	SYN	1663.1	−18.05	SYN
	CA103	>512	>512	32	1	0.06	SYN	1196.35	−9.63	SYN
	CA137	>512	>512	32	1	0.06	SYN	1089.68	−9.77	SYN
	CA632	>512	>512	32	1	0.06	SYN	1100.53	−3.94	SYN
	CA20003	>512	>512	32	0.5	0.06	SYN	1101.23	−3.92	SYN
LAN	CA10	>512	>512	32	1	0.06	SYN	1244.77	−0.61	SYN
	CA16	>512	>512	32	1	0.06	SYN	1437.05	−66.34	SYN
	CA103	>512	>512	32	1	0.06	SYN	1079.33	−15.94	SYN
	CA137	>512	>512	16	1	0.03	SYN	816.71	−7.27	SYN
	CA632	>512	>512	32	1	0.06	SYN	1249.21	−60.38	SYN
	CA20003	>512	>512	32	1	0.06	SYN	1103.44	−44.94	SYN
PTP	CA10	>512	>512	64	1	0.13	SYN	1341.94	−8.84	SYN
	CA16	>512	>512	64	1	0.13	SYN	1653.07	−24.73	SYN
	CA103	>512	>512	32	1	0.06	SYN	1106.52	−95.84	SYN
	CA137	>512	>512	32	1	0.06	SYN	823.01	−64.51	SYN
	CA632	>512	>512	64	1	0.13	SYN	1177.14	−63.02	SYN
	CA20003	>512	>512	64	1	0.13	SYN	1057.08	−53.32	SYN
RAB	CA10	256	>512	16	1	0.06	SYN	1149.42	−27.13	SYN
	CA16	256	>512	16	1	0.06	SYN	1157.63	−9.11	SYN
	CA103	256	>512	32	2	0.13	SYN	1044.78	−79.34	SYN
	CA137	256	>512	16	2	0.07	SYN	876.45	−74.15	SYN
	CA632	512	>512	16	4	0.04	SYN	1168.12	−163.45	SYN
	CA20003	128	>512	32	2	0.25	SYN	1235.58	−130.41	SYN
ESO	CA10	>512	>512	32	1	0.06	SYN	1334.34	−18.4	SYN
	CA16	>512	>512	32	1	0.06	SYN	1316.71	−1.07	SYN
	CA103	>512	>512	32	1	0.06	SYN	1178.25	−83.86	SYN
	CA137	>512	>512	32	1	0.06	SYN	1146.03	−49.13	SYN
	CA632	>512	>512	32	1	0.06	SYN	1062.42	−18.3	SYN
	CA20003	>512	>512	32	1	0.06	SYN	1086.97	−26.40	SYN
ILA	CA10	>256	>512	8	1	0.03	SYN	1072.23	−5.44	SYN
	CA16	>256	>512	8	1	0.03	SYN	1201.75	−10.62	SYN
	CA103	>256	>512	16	1	0.06	SYN	944.53	−95.54	SYN
	CA137	>256	>512	8	1	0.03	SYN	1045.04	−7.57	SYN
	CA632	>256	>512	8	2	0.04	SYN	953.47	−151.16	SYN
	CA20003	>256	>512	8	1	0.03	SYN	1306.81	−47.30	SYN

^a^PPIs: Proton pump inhibitors; IN, interpretation; SYN, Synergism; OME, omeprazole; LAN, Lansprazole; PTP, Pantoprazole; RAB, Rabeprazole; ESO, Esomeprazole; ILA, Ilaprazole;

^b^CA, *Candida albicans*;

^c^The MIC_80_ MIC was defined as the lowest concentration showing 80% growth inhibition; FICI, fractional inhibitory concentration index; ΣSYN and ΣANT were the sums of the percentages of all statistically significant synergistic and antagonistic interactions; MIC_80_ values, FICIs, ΣSYN and ΣANT are the median of three independent experiments.

### PPIs synergized with FLC against preformed biofilms of CA10

The sessile minimum inhibitory concentrations (sMIC) of PPIs and FLC against a resistant *C*. *albicans* strain (CA10) are listed in Table 2, and the data were analysed by the FICI model. For the biofilms preformed over 4, 8 and 12 h, the sMIC_80_ of FLC decreased significantly from >1024 μg/mL to 1–4 μg/mL, with the FICI values 0.06–0.13 for OME and RAB and 0.06–0.25 for LAN, PTP, ESO and ILA. The FICI values were all <0.5, showing strong synergistic antibiofilm effects induced by PPIs and FLC. When combined with PPIs against biofilms preformed over 24 h, there was almost no change in the sMIC_80_ of FLC compared with that of FLC alone, indicating an indifferent interaction between PPIs and FLC.

**Table 2:** *In vitro* interactions of PPIs with FLC against CA10 preformed biofilms.

PPIs^a^	Time (h)^b^	sMIC_80_ of drugs (µg/mL)^c^				FICI^c^	IN^a^
		Alone		Combined			
		PPIs	FLC	PPIs	FLC		
OME	4	>1024	>1024	64	1	0.06	SYN
	8	>1024	>1024	64	2	0.06	SYN
	12	>1024	>1024	128	4	0.13	SYN
	24	>1024	>1024	>1024	>1024	2	IND
LAN	4	>512	>1024	32	2	0.06	SYN
	8	>512	>1024	64	1	0.13	SYN
	12	>512	>1024	128	2	0.25	SYN
	24	>512	>1024	>512	>1024	2	IND
PTP	4	>1024	>1024	64	2	0.06	SYN
	8	>1024	>1024	128	2	0.13	SYN
	12	>1024	>1024	256	2	0.25	SYN
	24	>1024	>1024	>1024	>1024	2	IND
RAB	4	>1024	>1024	32	1	0.03	SYN
	8	>1024	>1024	64	1	0.06	SYN
	12	>1024	>1024	128	2	0.13	SYN
	24	>1024	>1024	>1024	>1024	2	IND
ESO	4	>1024	>1024	64	1	0.06	SYN
	8	>1024	>1024	64	2	0.06	SYN
	12	>1024	>1024	256	4	0.25	SYN
	24	>1024	>1024	>1024	>1024	2	IND
ILA	4	>256	>1024	16	1	0.06	SYN
	8	>256	>1024	32	2	0.13	SYN
	12	>256	>1024	64	1	0.25	SYN
	24	>256	>1024	>256	>1024	2	IND

^a^PPIs: Proton pump inhibitors; IN, interpretation; IND, Indifference; SYN, Synergism; OME, omeprazole; LAN, Lansprazole; PTP, Pantoprazole; RAB, Rabeprazole; ESO, Esomeprazole; ILA, Ilaprazole;

^b^Time, incubation period of preformed biofilm;

^c^The sMIC_80_ MIC was defined as the lowest concentration showing 80% biofilm metabolic activity inhibition; FICI, fractional inhibitory concentration index; sMIC_80_ values and FICIs are the median of three independent experiments.

### PPIs enhanced the efficacy of FLC against resistant *C*. *albicans in vivo*

**Figure 2:**
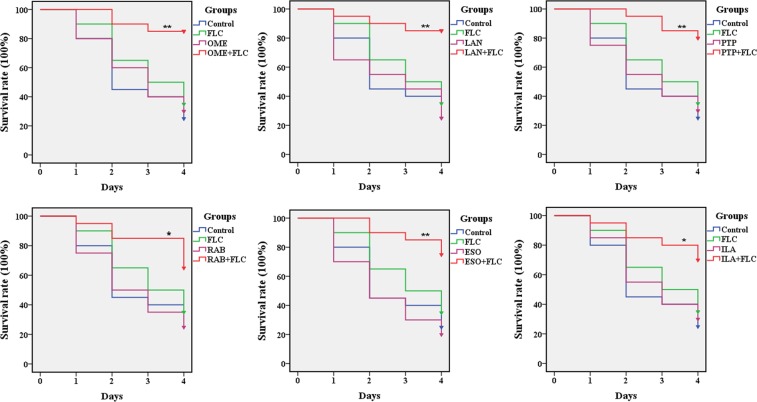
Survival rate of infected *G*. *mellonella* larvae treated with different drugs. After infection with CA10 (5 × 10^8^ CFU/mL), larvae were treated with PBS, FLC (160 μg/mL), OME (80 µg/mL), LAN (80 µg/mL), PTP (160 µg/mL), RAB (40 µg/mL), ESO (80 µg/mL), ILA (40 µg/mL) or PPIs plus FLC (160 μg/mL). The log-rank test was performed, and results were compared with the FLC-treated group; *P < 0.05, **P < 0.01.

In an *in vivo* experiment, 20 randomly chosen larvae in each group were injected with a *C*. *albicans* suspension, and after 2 h of infection, the larvae were treated with drugs. Regarding the survival rate of *G*. *mellonella* larvae (Fig. 2), 25% of the larvae in the control group survived until the end of observation period. With the monotherapy of FLC and PPIs, the survival rates of larvae were 20–35%, similar to that of the control group, indicating no significant antifungal effect of drug monotherapy on the larvae. Notably, PPIs combined with FLC kept the larvae free from *C*. *albicans* infections and resulted in 70–85% survival of the larvae over a 4-day infection. More specifically, the survival rates of the drug combination groups were 85% for OME and LAN, 80% for PTP, 75% for ESO, and 70% for RAB and ILA, demonstrating that the combination of PPIs and FLC significantly increased the survival rates of infected larvae (*P* < 0.05).

**Figure 3:**
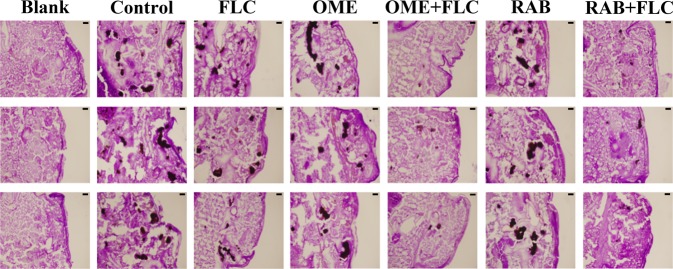
Histopathology of infected *G*. *mellonella* larvae treated with different drugs. After infection with CA10 (5 × 10^8^ CFU/mL), larvae were treated with PBS, FLC (160 μg/mL), OME (80 µg/mL), RAB (40 µg/mL), OME (80 µg/mL) plus FLC (160 μg/mL) or RAB (40 µg/mL) plus FLC (160 μg/mL). The larvae of the blank groups were not treated with yeast or any drugs. Tissue sections were observed at a 4.2 × 10 multiplier, with a scale of 20 µm.

Regarding observation of histological sections (Fig. 3), integrated and dense tissue was observed in the blank group with no black mass and uniform staining. In other groups, the infected tissues showed black lumps after PAS staining, and the lumps contained yeast cells and hyphae. More specifically, black lumps in the FLC-monotherapy group and PPI-monotherapy groups as well as the control group were numerous and large, while those in the combination treatment groups were obviously much fewer and smaller. These observations suggested that compared with the FLC monotherapy, PPIs combined with FLC significantly reduced the damage of the resistant *C*. *albicans* to the larvae.

### PPIs plus FLC synergistically suppressed the morphology switching of CA10

**Figure 4:**
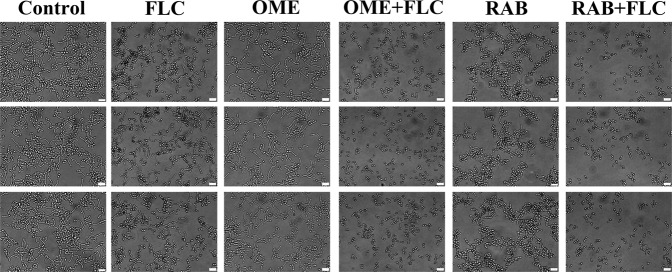
Effects of PPIs combined with FLC on the morphologic transformation of resistant *C*. *albicans*. A yeast suspension (5 × 10^5^ CFU/mL) was incubated in RPMI-1640 medium with PBS, FLC (8 µg/mL), OME (128 µg/mL), OME (128 µg/mL) plus FLC (8 µg/mL), RAB (64 µg/mL) or RAB (64 µg/mL) plus FLC (8 µg/mL). After an incubation of 5 h at 35 °C, cells were observed under an inverted microscope at a 40 × 10 multiplier, and the scale in the figure is 20 µm.

The hyphae growth of CA10 after the drug treatments was observed to evaluate the effect of the drug combination on the morphologic transformation of the resistant *C*. *albicans*. The results showed (Fig. 4) that the length of hyphae in the FLC and RAB monotherapy groups was much shorter than that in the control group and the OME monotherapy group, indicating a weak inhibitory effect induced by FLC and RAB but not by OME. Importantly, compared with that in the control group and the drug monotherapy groups, the length of hyphae in the drug combination groups, namely OME + FLC and RAB + FLC, was visibly shorter. Therefore, the drug combination could inhibit filamentous growth of drug-resistant *C*. *albicans* cells, and the inhibitory effects were higher than that of FLC alone.

### PPIs plus FLC synergistically inhibited the phospholipase activity of CA10

The P_Z_ value was calculated as P_Z_ = the diameter of colony/the diameter (colony + precipitation zone) to evaluate the phospholipase activity of *C*. *albicans*, and the higher the P_Z_ value was, the lower the phospholipase activity. In this study, very high phospholipase activity was observed in the control group and drug monotherapy group with P_Z_ values of 0.64–0.66 (Table 3). For the combination groups, the P_Z_ values were 0.87 ± 0.01 for OME plus FLC and 0.84 ± 0.01 for RAB plus FLC, showing a significantly lower phospholipase activity than that in other groups (*P* < 0.0001). These data indicated that PPIs combined with FLC could synergistically decrease the phospholipase activity of resistant *C*. *albicans*.

**Table 3:** Phospholipase activity of resistant *C*. *albicans* (CA10) treated with drugs.

Drugs^a^	P_z_ value ± SD^b^	Phospholipase activity
No drug	0.64 ± 0.02	Very high
FLC	0.66 ± 0.02^n.s^	Very high
OME	0.65 ± 0.01^n.s^	Very high
RAB	0.66 ± 0.01^n.s^	Very high
OME + FLC	0.87 ± 0.01****	Low
RAB + FLC	0.84 ± 0.01****	Low

^a^FLC, fluconazole (1 μg/mL); OME, omeprazole (32 μg/mL); RAB, rabeprazole (16 μg/mL);

^b^*P*_*z*_ values were the median of three independent experiments; Pz ≤ 0.69, very high phospholipase activity, Pz = 0.70–0.79, high activity; Pz = 0.80–0.89, low activity; Pz = 0.90–0.99, very low activity; Pz = 1, negative activity; SD, standard deviation; Compared with the control group, ^n.s^ *P* > 0.05; Compared with the control group and drug monotherapy groups; ****P < 0.0001.

### PPIs inhibited the efflux pump activity of CA10

**Figure 5:**
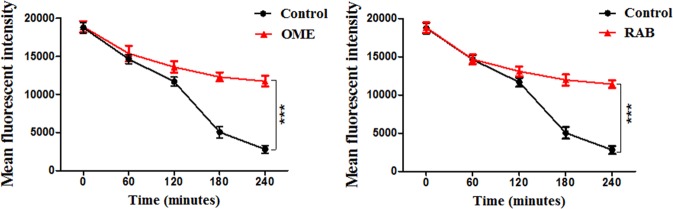
Inhibitory effects of PPIs on the efflux of R6G in resistant *C*. *albicans*. Fluorescent intensity was detected after the treatment with OME (128 μg/mL) and RAB (64 μg/mL) over 240 min. The statistical significance was determined by Student’s *t*-test and compared with the control group; ***P < 0.001.

The fluorescent dye rhodamine 6 G (Rh6G) and FLC both are substrates of drug transporters in *C*. *albicans*. Therefore, in this assay, we used Rh6G as a tracer of FLC to detect the intracellular FLC concentration. As shown in Fig. 5, the initial values of the MFI in the control group and the PPI-treated groups were almost the same, and the MFI showed a decreasing trend over time. Within 120 min, there was no difference between the MFI of control group and the PPI-treated groups. However, after 240 min, the MFI of the control group decreased significantly, while that of the PPI-treated groups decreased slightly. This result indicated that the addition of PPIs could inhibit the efflux pump activity of resistant *C*. *albicans*.

## Discussion

In recent years, the resistance of *C*. *albicans* to current antifungal agents has emerged frequently, and to solve this problem, research on sensitizers to existing antifungal agents or new antifungal drugs has received wide attention. Numerous studies on sensitizers to antifungal agents found that antibacterials^18^, calcium channel blockers^19^, phytocompounds^20^, etc., could enhance the sensitivity of resistant *C*. *albicans* to antifungals.

As the most potent acid-suppressing drugs, PPIs are considered as the standard treatment for acid-related diseases. Although acid suppression therapy has been reported as a risk factor for *Candida* oesophagitis^21^, PPIs represent an opportunity for joint applications with FLC for *Candida* oesophagitis in the clinic. For example, Hasosah, M. Y. et *al*. and Liang, M. et *al*. reported that immunocompetent patients presenting with *Candida* oesophagitis were successfully cured by OME combined with FLC^22,23^. Studies on the antifungal interaction of PPIs and FLC have also been conducted previously, and some studies showed antagonistic effects against susceptible *C*. *albicans* isolates^13,14^. However, there was no report about whether PPIs could increase the sensitivity of resistant *C*. *albicans* to FLC. Based on these studies, we tested the interaction of PPIs combined with FLC against both susceptible and resistant *C*. *albicans* strains. In a preliminary experiment, we observed a strange phenomenon for all the drug combinations against the susceptible strain. The combination of FLC and RAB serves as an example to clarify this phenomenon. As shown in Table S1, compared with 0.25 μg/mL FLC alone, the addition of 4–32 μg/mL RAB showed a stronger inhibitory effect on the *C*. *albicans* susceptible strain CA4; however, a higher concentration of RAB (64–128 μg/mL) resulted in a weaker inhibitory effect. To explain this phenomenon, many experiments need to be done. Therefore, we focused only on the resistant strain in this study. More importantly, Monk, B. C. *et al*. and Hayama, K. *et al*. found that an inhibitor of the plasma membrane proton pump (BM2) could enhance the efficacy of FLC against resistant *C*. *albicans* and *C*. *dubliniensis*^16^. Inspired by the synergism of BM2 with FLC, we evaluated the interaction of PPIs combined with FLC against resistant *C*. *albicans* and further explored the underlying mechanisms.

*In vitro*, we found that PPIs exerted weak anticandidal effects with MICs ≥ 128 μg/mL, which was in accordance with the studies of Biswas, S. K. *et al*. and Liu, N *et al*.^12,13^. More importantly, PPIs all acted synergistically with FLC against six tested resistant *C*. *albicans* isolates, as interpreted by the FICI and Δ*E* model. These six PPIs decreased the MIC_80_ of FLC from > 512 μg/mL to 0.5–4 μg/mL. Among them, OME reduced the MICs of FLC to a maximum extent, while RAB reduced the MICs of FLC to a minimum extent. Additionally, when the MIC of FLC was decreased to the same concentration, the minimum concentration of proton pump inhibitors required is 8 µg/mL for ILA, and the maximum is 32–64 µg/mL for PTP, demonstrating that different PPIs resulted in different enhanced efficacy of FLC.

Biofilm formation has been shown to be related with drug resistance of *C*. *albicans*^24^. In clinic, biofilm-related infections are difficult to treat due to their trend to be chronic and easy recurrence. Here, the sMIC_80_ of FLC for resistant *C*. *albicans* biofilms preformed for ≤12 h was decreased from >1024 µg/mL to 1–4 µg/mL by the presence of PPIs, indicating enhancement of the antibiofilm effect of FLC by PPIs. In addition, different PPIs resulted in different enhanced efficacy of FLC. With prolonged preformation time, the biofilm was more mature, and the synergistic effect was weaker. The biofilm preformed for 24 h was much more mature and its structure was more complex. Therefore, no obvious inhibitory action induced by the drug combinations was observed. Studies on the antibiofilm effect of drugs also showed that drugs are less effective in mature *C*. *albicans* biofilms than in early-stage biofilms and even not effective in mature biofilms^25,26^. Our findings demonstrated a potential use of the drug combination in prevention or early treatment of biofilm-related diseases.

The *G*. *mellonella* larva is a type of infection model that has been used to study the efficacy or toxicity of drugs as well as the virulence of pathogens^27,28^. Compared with mammal host models, this infection model can provide a more rapid evaluation of the virulence of pathogens and the *in vivo* efficacy or toxicity of agents, with significant economic and ethical advantages^29,30^. To primarily assess the combined effects of PPIs and FLC *in vivo*, we used this model to determine survival rates of the larvae infected with CA10. The data obtained showed that the survival rates of the larvae treated with PPIs + FLC were significantly higher than those of the FLC monotherapy group and other groups, demonstrating that the tested PPIs could significantly increase the *in vivo* efficacy of FLC against resistant *C*. *albicans*. Histopathological study of *G*. *mellonella* larvae was carried out to further assess the combined effects of PPIs plus FLC *in vivo*. In this study, the tissue infected with CA10 was stained as black lumps and was much more fragmentary than that of the blank group. This result indicated that the resistant *C*. *albicans* CA10 could cause serious damage to the tissues of the larvae. In addition, fewer and smaller black lumps were observed in the drug combination group than in the control group and the drug-monotherapy groups, demonstrating that at the experimental concentration, the drug combinations could suppress the damage caused by resistant *C*. *albicans* to the larvae. Taken together, these data show that PPIs could significantly enhance the *in vivo* efficacy of FLC against resistant *C*. *albicans*, which was in accordance with the *in vitro* results.

*C*. *albicans* can switch among different morphological phenotypes, and the morphology switching is a primary virulence factor^31,32^. Notably, this switch has been proven to be involved in the pathogenicity and biofilms formation of *C*. *albicans*^33,34^. Here, we found that RAB possessed a weak inhibitory effect on the morphology switching of both CA10 and a susceptible *C*. *albicans* strain (CA4) (Fig. S1). More importantly, compared with FLC monotherapy, PPIs combined with FLC possessed a stronger inhibitory effect on hyphae formation. This finding demonstrated that the inhibition of the morphology switching might be a mechanism of the synergistic antifungal effect induced by PPIs combined with FLC. In addition, LAN was also found to possess a weak inhibitory effect on the hyphae formation of CA4 (Fig. S2), and this finding was coincident with earlier research, which reported the inhibition of hyphal growth of *C*. *albicans* by LAN.

Phospholipase, as one of the most important hydrolase of *C*. *albicans*, is another important virulence factor in *C*. *albicans* infections^35,36^. Ying *et al*. found that the phospholipase B1 mRNA and protein expression of resistant strains was higher than that of susceptible strains^37^. In addition, *C*. *albicans* phospholipase D1 has been proven to play a role in promoting the transformation of yeast to mycelium^38^. These studies demonstrated that the phospholipase activity of *C*. *albicans* might be related to the resistance and morphology switching. In the present study, the phospholipase activities of *C*. *albicans* in the PPIs plus FLC groups were significantly reduced, with P_z_ values higher than 0.80. However, drug combinations at the same concentrations could not decrease the phospholipase activity of a susceptible *C*. *albicans* (CA4) (Table S3). Inspired by the Ying *et al*. study^37^, we determined that the synergism of PPIs and FLC against resistant *C*. *albicans* might be related to phospholipase B1, and this observation needs further study.

As one of the most common resistance mechanisms in *C*. *albicans*, overexpression of efflux pumps has become a research hotspot for *Candida* resistance^39,40^. The present study found that compared with the control group, the addition of PPIs significantly suppressed efflux pump activity (*P* ˂ 0.001). Additionally, Monk, B. C. *et al*. and Hayama, K. *et al*. found that the inhibition of efflux pumps may be involved in the enhanced efficacy of FLC to resistant *C*. *albicans* and *C*. *dubliniensis* caused by a D-octapeptide inhibitor of the plasma membrane proton pump (BM2)^16,17^. These findings demonstrated that the synergism between PPIs and FLC may be related to efflux pumps suppression.

In conclusion, this paper provides an advance over our recent studies and in the field by first finding that PPIs enhanced the efficacy of FLC against resistant *C*. *albicans* both *in vitro* and *in vivo* and that the efflux pump suppression, extracellular phospholipase inhibition and morphology switching suppression may be involved in the synergistic antifungal effects. These findings together with the opportunity for the combined applications of PPIs and FLC in the clinic will provide new insights into antifungal agent discovery and potential approaches for the treatment of candidiasis caused by resistant *C*. *albicans*.

## Methods and Materials

### Strains

The six resistant *C*. *albicans* strains used in this study are listed in Table 1. The first three in the table were collected from the clinical laboratory at Shandong Provincial Qianfoshan Hospital (Ji’nan, China) and the other three were kindly provided by Professor Changzhong Wang (School of Integrated Traditional and Western Medicine, Anhui University of traditional chinese medicine, Hefei, China). *C*. *albicans* ATCC10231 was used as a quality control strain to determine the MICs of drugs and was provided by the Institute of Pharmacology, School of Pharmacy, Shandong University (Ji’nan, China). Strains were stored at −80 °C in the Sabouraud dextrose broth and subcultured on the Sabouraud dextrose agar for 24 h at 35 °C before the experiment. Egg yolk agar (0.01 M NaCl, 0.025 M CaCl_2_, 1% peptone, 3% glucose, 2% agar and 10% egg yolk) was used to test the phospholipase activity.

### Drugs

PPIs and FLC were all purchased from Dalian Meilun Biotech Co., Ltd. (Liaoning, China). Stock solutions of OME, PTP, RAB and ESO were prepared in sterile distilled water, while those of FLC, LAN and ILA were prepared in dimethyl sulfoxide. All stock solutions were sterilized using 0.22-μm filters, and that of FLC was stored at 4 °C while stock solutions of PPIs were prepared before each experiment. Rhodamine 6 G was purchase from Acros Trading Company.

### Antifungal susceptibility testing

The antifungal activities of PPIs and FLC against the six resistant *C*. *albicans* were tested by the broth microdilution method according to the Clinical and Laboratory Standards Institute (CLSI) guidelines. The test was conducted in 96-well microtiter plates with yeast (2.5 × 10^3^ CFU/mL) in RPMI-1640 medium (PH 7.0) buffered with MOPS [morpholino (propanesulfonic acid)]. Wells containing only RPMI-1640 medium served as negative controls, and a drug-free well was set as a growth control. After 24 h of incubation at 35 °C, the MICs were determined by both visual reading and measuring the optical density (OD) with a microplate reader at a wavelength of 492 nm. The MIC_80_ was defined as the lowest concentration of drug with 80% fungal growth inhibition.

### Checkerboard microdilution assay

A checkerboard microdilution assay was carried out to determine the interactions between PPIs and FLC against resistant *C*. *albicans*. Briefly, drugs were serially diluted 2-fold in RPMI-1640 medium, and 0.25–128 μg/mL FLC, 2–128 μg/mL OME, LAN, PTP, RAB and ESO and 0.5–32 μg/mL ILA were added to the wells. Subsequently, yeast at a final concentration of 2.5 × 10^3^ CFU/mL was added to each well. Wells containing only RPMI-1640 medium served as negative controls, and a drug-free well was set as a growth control. After 24 h of incubation at 35 °C, MICs were determined as described above.

To evaluate the mode and intensity of the drug interactions, the fractional inhibitory concentration index (FICI) model and the Δ*E* model were used to analyse the obtained data. The FICI model is based on the Loewe additivity theory^41^ and is expressed as FICI = FIC_A_ + FIC_B_ = $\frac{{{\rm{MIC}}}_{{\rm{A}}}^{{\rm{Comb}}}}{{{\rm{MIC}}}_{{\rm{A}}}^{{\rm{Alone}}}}+\frac{{{\rm{MIC}}}_{{\rm{B}}}^{{\rm{Comb}}}}{{{\rm{MIC}}}_{{\rm{B}}}^{{\rm{Alone}}}}$. In this model, the drug interaction is interpreted as synergistic when FICI ≤ 0.5, indifferent when FICI > 0.5–4.0, and antagonistic when FICI > 4.0. The Δ*E* model is based on the Bliss independence theory^42^ and is expressed as Δ*E* = *E*_A_ × *E*_B_ − *E*_measured_. In this equation, *E*_A_ and *E*_B_ are the experimental fungal growth percentages when each drug acts alone, and *E*_measured_ is the measured growth percentage in presence of the combination of drugs A and B. The mode of interaction is interpreted as synergistic when Δ*E* and its 95% confidence interval (CI) are positive, antagonistic when Δ*E* and its 95% CI are negative, and indifferent in other cases. The intensity of interaction is evaluated by calculating the sum percentages of all significant synergistic (ΣSYN) or antagonistic (ΣANT) interactions and is interpreted as strong when interactions are >200%, moderate when interactions are 100–200%, and weak when interactions are <100%.

### Antibiofilm assay

The interactions between PPIs and FLC against preformed biofilms of *C*. *albicans* (CA10) in different growth periods were assessed as previously described, with slight modification^43^. Briefly, 200-μL aliquots of a yeast suspension (2.5 × 10^3^ CFU/mL) were added to a 96-well plate, and the plates were incubated for four different time intervals (4, 8, 12 and 24 h) at 35 °C to preform biofilms. Then, the preformed biofilms were washed three times with sterile phosphate-buffered saline (PBS), and drugs were added at final concentrations of 1–1024 μg/mL FLC, 16–1024 µg/mL OME, PTP, RAB and ESO, 8–512 µg/mL LAN and 4–256 µg/mL LAN. Following a further 24 h of incubation at 35 °C, an XTT reduction assay was performed to examine the metabolic activity of the biofilms. Colorimetric changes were measured with a microplate reader at a wavelength of 492 nm. The sessile minimum inhibitory concentration (sMIC) was defined as the lowest concentration of drug with 80% inhibition of biofilm metabolic activity.

### *In vivo* infection model

*Galleria mellonella* larvae were used as an *in vivo* infection model to evaluate the *in vivo* interactions between PPIs and FLC, and CA10 was used to infect the *G*. *mellonella* larvae^44,45^. For the survival assay, fourteen groups of 20 randomly chosen larvae with a similar size (ca. 0.25 g) and no grey markings were selected and injected with 10 μL of a yeast suspension (5 × 10^8^ CFU/mL) *via* the last left proleg. After 2 h of incubation at 35 °C, the larvae were injected with 10 μL of sterile PBS, 160 μg/mL FLC, PPIs, or PPIs + 160 μg/mL FLC *via* the last right proleg. The concentration of PPIs was 40 μg/mL RAB and ILA, 80 μg/mL OME, LAN and ESO and 160 μg/mL PTP. Then, the larvae were placed in the dark and incubated at 35 °C for 4 days. The survival rate of the larvae was monitored daily, considering death of the larvae when they did not respond to physical pressure. For histological study, OME and RAB were selected to evaluate the interactions between PPIs and FLC on the tissue of infected larvae. Seven groups of larvae were selected and injected with a yeast suspension and drugs as described above. One group of larvae untreated with the yeast and drugs served as a blank control group. After a further 48-h incubation, three larvae from each group were randomly selected and cut into sections (20 μm). Sections stained with periodic acid Schiff (PAS) stain were observed under a fluorescence microscope.

### Yeast-to-hyphae morphogenesis

Effects of PPIs combined with FLC on the yeast-to-hyphae morphogenesis of resistant *C*. *albicans* (CA10) were studied in a microplate-based assay^46^. In this assay, OME was selected as the representative PPI, and yeast-to-hyphae morphogenesis was induced by RPMI-1640. Yeast cells (10^5^ CFU/mL) were added to a 6-well microplate, and drugs were then added at the final concentration of 8 μg/mL FLC, 128 μg/mL OME and 64 μg/mL RAB. The microplate was incubated at 35 °C for 5 h and then was placed directly under an inverted microscope.

### Extracellular phospholipase activity assay

Effects of PPIs combined with FLC on the extracellular phospholipase activity of resistant *C*. *albicans* (CA10) were detected by egg yolk agar plates^47^, and OME and RAB were selected as representative PPIs. Yeast cells (10^6^ CFU/mL) were incubated with no drug, FLC (1 μg/mL), OME (32 μg/mL), RAB (16 μg/mL), OME (32 μg/mL) plus FLC (1 μg/mL), or RAB (16 μg/mL) plus FLC (1 μg/mL) for 24 h at 35 °C. After the incubation, 10 μL of the cell suspensions were inoculated onto egg yolk agar plates and the plates were then incubated for 72 h at 35 °C. The colony diameter and precipitation zone diameter were measured.

### Efflux pump assay

Whether PPIs interfered with the efflux pump activity of resistant *C*. *albicans* was evaluated by the Rh6G efflux assay with some modification^48^, and a resistant isolate with an efflux pump gene over-expressed (CA10) was used. Briefly, yeast cells were incubated in YPD liquid medium overnight, and the cells were harvested, washed with glucose-free PBS and adjusted to 10^7^ CFU/mL. Subsequently, a Rh6G solution was added at a final concentration of 10 μM to the cell suspension, and the suspension was incubated at 35 °C for 50 min and then exposed to an ice water-bath for 10 min. Cells were harvested and washed with glucose-free PBS. Glucose/PBS (5%) was added to re-suspend the cells, and PPIs were added. The fluorescence intensity was detected every 60 min for 4 h by flow cytometry, with excitation at 488 nm and emission at 530 nm.

### Statistical analysis

Each experiment was performed three times on different days. Graphs were produced with GraphPad Prism 5 and MATLAB 2017a, and statistical analyses were performed with SPSS Statistics v.17.0. The survival curve was analysed by the Kaplan-Meier method and log-rank test. *P* values < 0.05 were considered statistically significant.

## Supplementary information

Supplementary Information.

## Data Availability

All data are shown in the paper. Other datasets generated during and/or analyzed during the current study would be provided on reasonable request.
