# Supplementary material for: Proton pump inhibitors act synergistically with fluconazole against resistant Candida albicans
Source: Sci Rep. 2020 Jan 16;10:498. doi: 10.1038/s41598-019-57174-4 (PMC6965112; doi:10.1038/s41598-019-57174-4)
Supplement: Supplementary file 1 — Supplementary Information. [file 41598_2019_57174_MOESM1_ESM.pdf]

Proton pump inhibitors act synergistically with fluconazole against resistant *Candida albicans*

Mengjiao Lu[1,2], Haiying Yan[3], Cuixiang Yu[4], Lei Yuan[2], Shujuan Sun[3*]

[1]Department of Pharmacy, Shandong Provincial Qianfoshan Hospital, Shandong University, Ji'nan, 250014, Shandong Province, P.R. China;
[2]Department of Pharmacy, Tianjin Baodi Hospital, Baodi Clinical College of Tianjin Medical University, Tianjin, 301800, P.R. China;
[3]Department of Pharmacy, Shandong Provincial Qianfoshan Hospital, the First Hospital Affiliated with Shandong First Medical University, 250014, Shandong Province, P.R. China;
[4]Respiration Medicine, Shandong Provincial Qianfoshan Hospital, the First Hospital Affiliated with Shandong First Medical University, 250014, Shandong Province, P.R. China.

Correspondence to
Shujuan Sun, Department of Pharmacy,
Shandong Provincial Qianfoshan Hospital, the First Hospital Affiliated with
Shandong First Medical University
Ji'nan, 250014, P.R. China.
Tel: 86-531-89268365
Fax: 86-531-82961267
E-mail: sunshujuan888@163.com

Contents

Part 1 *In vitro* interaction of RAB combined with FLC against a susceptible *C. albicans* strain (CA4) P1

Part 2 Interaction of RAB combined with FLC and the effect of LAN on Yeast-to-Hyphae Morphogenesis of CA4 P2

Part 3 Interaction of OME and RAB combined with FLC on phospholipase activity of CA4 P5

Part 4 Relative expression efflux gene in CA10 P6

Part 1 *In vitro* interaction of RAB combined with FLC against a susceptible *C. albicans* strain (CA4)

Method: Checkerboard microdilution assay was carried out to determine the interactions of RAB and FLC against CA4. Briefly, drugs were serially diluted 2-fold in RPMI-1640 medium, and drugs at final concentrations of 0.03-16 μg/mL for FLC and 2-128 μg/mL for RAB were added to the wells. Subsequently, the yeast at a final concentration of 2.5×10^3 CFU/mL was added to each well. The wells containing RPMI-1640 medium acted as negative controls, and a drug-free well was set as the growth control. After 24 h of incubation at 35 ℃, MICs were determined as mentioned above.

Results: Results were presented as the **Table S1** and **Fig. S1**. As shown in Table **S1**, FLC at the concentration of 0.5μg/ml caused about 80% growth inhibition, indicating a MIC_{80} of FLC 0.5μg/ml. In addition, more than 80% growth inhibition was induced by 4-32 μg/ml RAB plus 0.25μg/ml FLC. However, when 64-128 μg/ml RAB combined with 0.25μg/ml FLC, the growth rate was more than 60%, showing an antagonism effect. This result was also observed in CA 8, another susceptible strain.

Table S1 Growth rate of a susceptible *C. albicans* strain CA4 treated with the combination of RAB and FLC

**Growth rate**		**FLC(μg/ml)**											
		0	0.03	0.06	0.13	0.25	0.5	1	2	4	8	16	Blank
**RAB(μg/ml)**	128	99%	96%	94%	91%	92%	74%	27%	0%	0%	0%	0%	0%
	64	98%	97%	95%	92%	62%	32%	7%	0%	1%	0%	0%	0%
	32	98%	97%	95%	35%	12%	7%	7%	3%	0%	0%	1%	0%
	16	98%	95%	94%	34%	5%	6%	7%	5%	3%	3%	3%	0%
	8	100%	100%	99%	38%	8%	7%	4%	5%	5%	4%	3%	0%
	4	100%	100%	100%	26%	9%	7%	4%	3%	4%	3%	3%	0%
	2	100%	99%	100%	23%	15%	5%	3%	3%	4%	5%	4%	0%
	0	100%	100%	97%	27%	27%	21%	7%	5%	4%	4%	4%	0%

Part 2 Interaction of RAB combined with FLC and the effect of LAN on Yeast-to-Hyphae Morphogenesis of CA4.

Method: Effect of RAB combined with FLC on the yeast-to-hyphae morphogenesis of a susceptible *C. albicans* (CA4) was studied in a microplate-based assay as the manuscript. In this assay, the yeast cells (10^5 CFU/mL) were added to a 96-well microplate, and drugs at the final concentrations of 2 μg/mL for FLC, 64-512 μg/mL for RAB and 128-1024 μg/mL for LAN were then added. The microplate was incubated at 35 ℃ for 5 h, and then was placed directly under the inverted microscope.

Results: Interaction of RAB combined with FLC on the yeast-to-hyphae morphogenesis of CA4 was shown in the **Fig. S1**, and the effect of LAN was shown in the **Fig. S2**. From the **Fig. S1**, RAB was found to inhibit hyphae formation when it was used alone, and the higher the dose, the shorter the hyphae. The length of hyphae in the 512 μg/mL RAB group was the shortest. However, when RAB combined with FLC, the shortest hyphae was observed in the group of 256 μg/mL RAB + FLC, but not 512 μg/mL RAB + FLC. This result accorded with that of *in vitro* interaction, indicating that yeast-to-hyphae morphogenesis was involved in the interaction of PPIs combined with FLC against *C. albicans*.

For the effect of LAN on the yeast-to-hyphae morphogenesis of CA4, **Fig. S2** showed that the hyphae in the control group were longest among all groups, and a large area of hyphae gathered together. In the LAN-treated groups, as the dose of LAN increased, the hyphae were getting shorter and less hypha gathered. Although there was no difference between LAN_{1024} and LAN_{512} groups in the length of hyphae, the number of cells in the LAN_{1024} group was less than that of LAN_{512} group. These findings demonstrated LAN might possess a weak inhibitory effect on the yeast-to-hyphae morphogenesis of susceptive *C. albicans* strains.

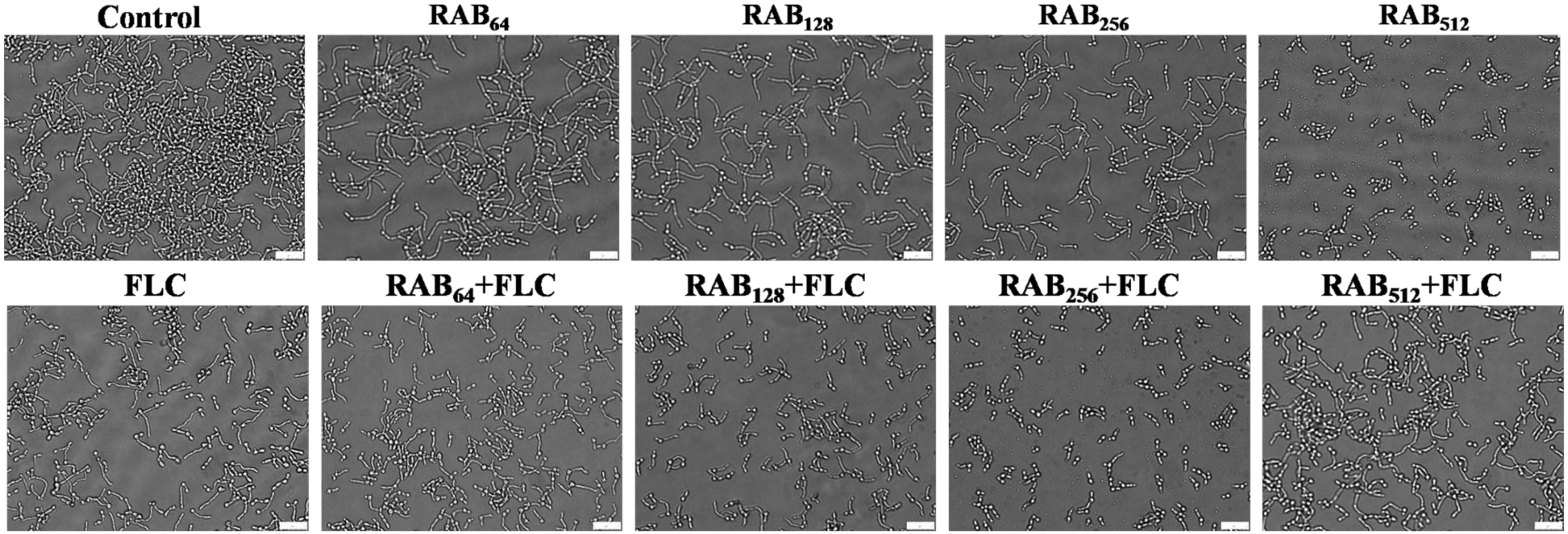

Fig. S1 Interaction of RAB combined with FLC on the yeast-to-hyphae morphogenesis of CA4. The yeast suspension (5×10^5 CFU/mL) were incubated in RPMI-1640 medium with PBS, FLC (2 μg/mL), RAB (64, 128, 256 and 512 μg/mL), RAB(64, 128, 256 and 512 μg/mL) plus FLC (2 μg/mL), respectively. After an incubation of 5 h at 35 ℃, cells were observed under the inverted microscope at a 40×10 multiplier, and the scale in the figure was 25 μm. RAB_{64}, RAB_{128}, RAB_{256} and RAB_{512} are RAB at the dose of 64, 128, 256 and 512 μg/mL, respectively.

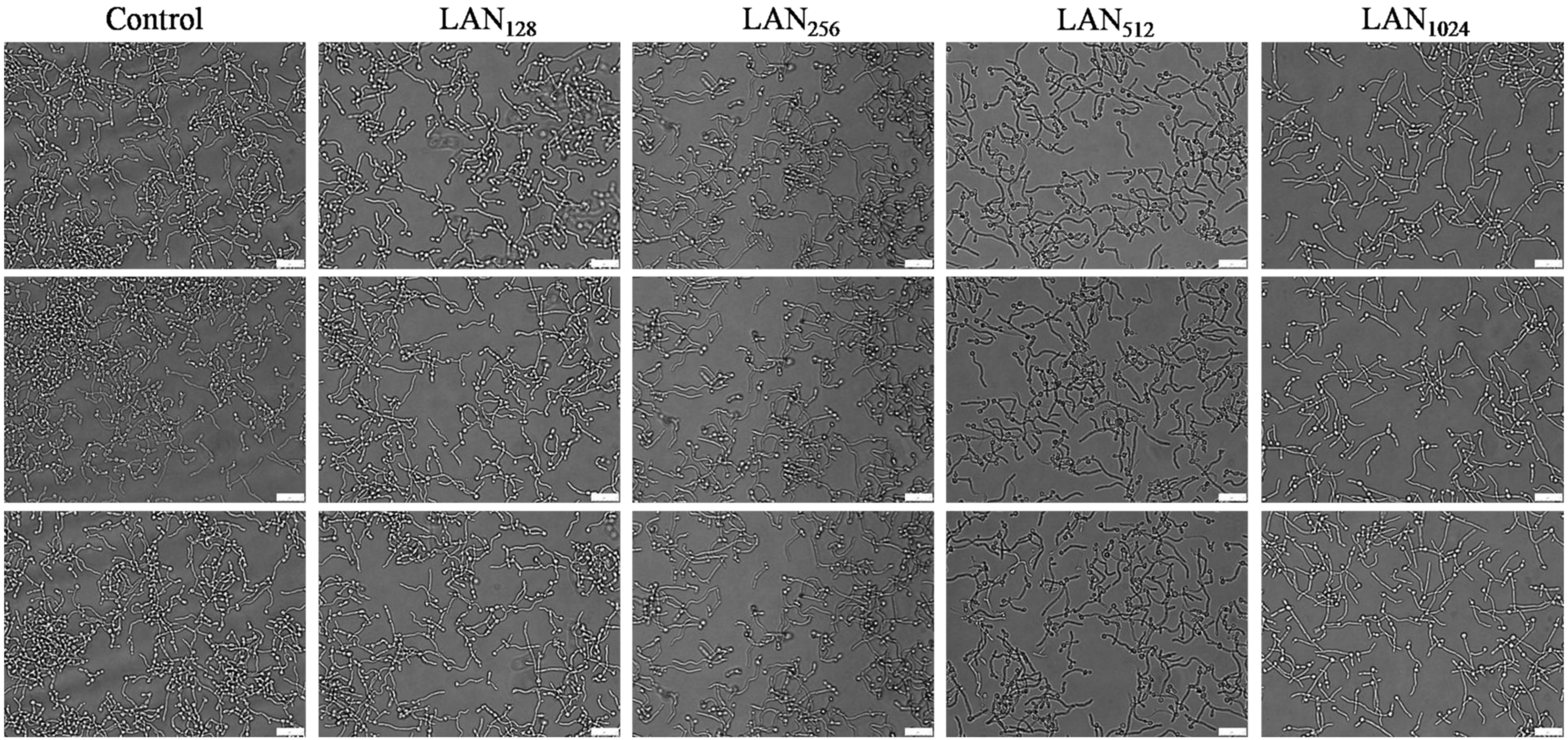

Fig. S2 Effect of LAN on the yeast-to-hyphae morphogenesis of CA4. The yeast suspension (5×10^5 CFU/mL) were incubated in RPMI-1640 medium with PBS, RAB (128, 256, 512 AND 1024 μg/mL), respectively. After an incubation of 5 h at 35 ℃, cells were observed under the inverted microscope at a 40×10 multiplier, and the scale in the figure was 25 μm. LAN_{128}, LAN_{256}, LAN_{512} and LAN_{1024} are LAN at the dose of 128, 256, 512 and 1024 μg/mL, respectively.

Part 3 Interaction of OME and RAB combined with FLC on phospholipase activity of CA4.

Methods: Effects of OME or RAB combined with FLC on the extracellular phospholipase activity of CA4 were detected by egg yolk agar plates. The yeast cells (10^6 CFU/mL) were incubated with no drug, FLC (1 μg/mL), OME (32 μg/mL), RAB (16 μg/mL), OME (32 μg/mL) plus FLC (1 μg/mL), RAB (16 μg/mL) plus FLC (1 μg/mL) for 24 h at 35 °C. After the incubation, 10 μL of the cell suspensions were inoculated onto egg yolk agar plates and the plates were then incubated for 72 h at 35 ℃. The colony diameter and precipitation zone diameter were measured.

Results: As shown in the **Table S2**, P_Z values of control group and drug-monotherapy groups were 0.65-0.66, showing a very high phospholipase activity. For the combination groups, the very high phospholipase activity was also observed with the P_Z values both 0.68±0.01. There was no difference between control and drug-monotherapy groups or drug combination groups ($P > 0.05$), demonstrating that drug monotherapy or drug combination has no inhibitory effect on the phospholipase activity of CA4.

Table S2 Phospholipase activity of CA4 treated with drugs

Drugs	P_z value ± SD[b]	Phospholipase activity
No drug	0.65±0.02	Very high
FLC	0.66±0.02[n.s]	Very high
OME	0.65±0.01[n.s]	Very high
RAB	0.66±0.02[n.s]	Very high
OME+FLC	0.68±0.01 [n.s]	Very high
RAB+FLC	0.68±0.01 [n.s]	Very high

[a] FLC, fluconazole (1 μg/mL); OME, omeprazole (32 μg/mL); RAB, rabeprazole (16 μg/mL).

[b] P_z values were the median of three independent experiments; Pz ≤ 0.69, very high phospholipase activity, Pz = 0.70-0.79, high activity; Pz = 0.80-0.89, low activity; Pz = 0.90-0.99, very low activity; Pz = 1, negative activity; SD, standard deviation; Compared with the control group, [n.s] $P > 0.05$.

Part 4 Relative expression efflux gene in CA10

Methods: The transcriptional expression of *CDR1*, *CDR2* and *MDR1* in the tested *C. albicans* SC5314 or CA10 was measured using qPCR as previously reported (PMID: 27503639). The primers used are shown in the Table S6. 18S rRNA served as the internal control in *C. albicans*. The transcript levels of the detected genes were calculated using the formula $2^{-\Delta\Delta CT}$.

Table S3 Primers used in this assay

Genes	Primer sequences (5'→3')
18S rRNA	F: AATTACCCAATCCCGACAC
	R: TGCAACAACTTTAATATACGC
CDR1	F: TAACACTTATGGTTTCCACAT
	R: AGCATAAGTTTCTCTGTCGA
CDR2	F: GAGTGTTGGTGATACTTTGG
	R: CACTCAAAGAAGCTTCAGCA
MDR1	F: AGATAATCAAGGTGAACCCAA
	R: GCTGATCCCATATAAACTGAA

Results: In this study, we measured the transcriptional expression of *CDR1*, *CDR2* and *MDR1* in both *C. albicans* SC5314 and CA10. *C. albicans* SC5314 was a wild isolate with no gene mutation. Compared with this wild isolate, gene expression of efflux pumps (*CDR1*, *CDR2* and *MDR1*) in CA10 was much higher ($P < 0.001$) (**Fig. S3**). In this case, the efflux pump was generally considered to be over-expressed.

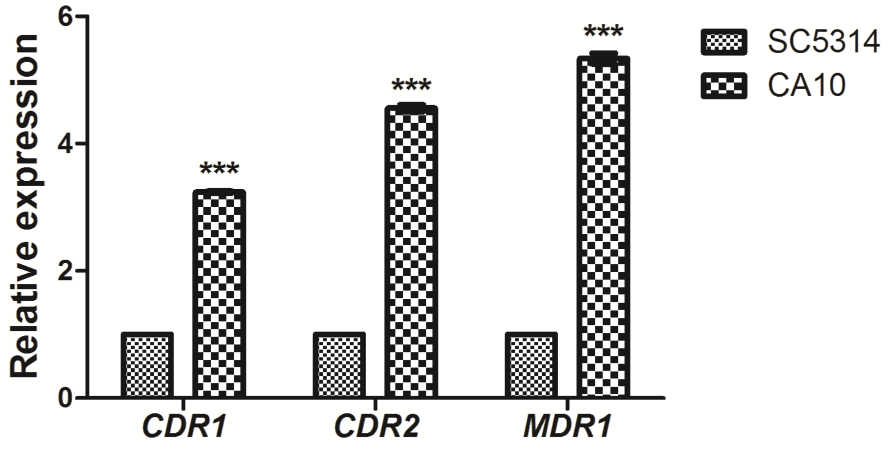

Fig. S4 Relative expression efflux gene in CA10. *C. albicans* SC5314 was a wild isolate with no gene mutation. Error bars indicated standard errors of the means. Statistical significances were determined by Student's t-test. $^{***}P < 0.001$ when compared with the respective controls.
